# Machine learning framework to segment sarcomeric structures in SMLM data

**DOI:** 10.1038/s41598-023-28539-7

**Published:** 2023-01-28

**Authors:** Dániel Varga, Szilárd Szikora, Tibor Novák, Gergely Pap, Gábor Lékó, József Mihály, Miklós Erdélyi

**Affiliations:** 1 Department of Optics and Quantum Electronics, University of Szeged, Dóm tér 9, Szeged, 6720 Hungary; 2 Institute of Genetics, Biological Research Centre, Temesvári körút 62, Szeged, 6726 Hungary; 3 Department of Computer Algorithms and Artificial Intelligence, University of Szeged, Árpád tér 2, Szeged, 6720 Hungary; 4 Department of Software Engineering, University of Szeged, Dugonics tér 13, Szeged, 6720 Hungary; 5 Department of Genetics, University of Szeged, Közép fasor 52, Szeged, 6726 Hungary

**Keywords:** Optical physics, Computational biology and bioinformatics, Physics

## Abstract

Object detection is an image analysis task with a wide range of applications, which is difficult to accomplish with traditional programming. Recent breakthroughs in machine learning have made significant progress in this area. However, these algorithms are generally compatible with traditional pixelated images and cannot be directly applied for pointillist datasets generated by single molecule localization microscopy (SMLM) methods. Here, we have improved the averaging method developed for the analysis of SMLM images of sarcomere structures based on a machine learning object detection algorithm. The ordered structure of sarcomeres allows us to determine the location of the proteins more accurately by superimposing SMLM images of identically assembled proteins. However, the area segmentation process required for averaging can be extremely time-consuming and tedious. In this work, we have automated this process. The developed algorithm not only finds the regions of interest, but also classifies the localizations and identifies the true positive ones. For training, we used simulations to generate large amounts of labelled data. After tuning the neural network’s internal parameters, it could find the localizations associated with the structures we were looking for with high accuracy. We validated our results by comparing them with previous manual evaluations. It has also been proven that the simulations can generate data of sufficient quality for training. Our method is suitable for the identification of other types of structures in SMLM data.

## Introduction

Single molecule localization microscopy (SMLM)^1–4^ has become a widely used and accepted tool in molecular cell biology research^5^. By utilizing the localization of single molecules, previously unseen spatial resolution ($\sim$ 10 nm) has been achieved in the optical regime^6^. The raw data provided by SMLM is a point cloud, i.e. a list of spatial coordinates of the localized emitters, which is fundamentally different from the pixelated images of conventional optical microscopes. Consequently, the interpretation, quantification and visualization of such data require new approaches and solutions. Conventional pixelated images can be generated from the localization data^7–11^, however such conversion introduces a loss of information^12–14^. Therefore, the direct extraction of the relevant information from the raw localization data requires extra effort. Another hurdle of the interpretation of SMLM measurements is its labour intensity. Data evaluation often requires the analysis of data belonging to many identical structures. Selecting the structures of interest and analyzing them individually is time consuming and tedious if performed manually. To this end, object classification^15^ and structure averaging^16^ methods have been developed and made public recently. Machine learning algorithms are gaining widespread attention for the analysis of complex data^17^. Sometimes, it is difficult to write an exact algorithm that the computer can follow to solve a specific task. In such cases, one option is to use machine learning methods. If we know the possible response signal of the system for a given input, supervised machine learning can be applied. Otherwise, without prior knowledge, non-supervised machine learning algorithms can be used to find patterns in the data or label data points. Artificial Neural Networks (ANNs) are widely used in supervised machine learning. They are made up of artificial neurons that can receive and process input data, and subsequently provide an output. Neurons with similar functions are grouped together to form layers. The machine tunes the internal parameters of the neural network based on a known training dataset so that its output converges to the expected output. The data generated by localization algorithms are not directly compatible with most neural network constructs used for image analysis, as they require an input of fixed size, which is less straightforward to achieve than with pixelated images. Attempts have been made to analyze localization data using machine learning^18–21^, however, the field is still highly unexploited. In this work, we have created a supervised machine learning workflow for the analysis of localization data, more specifically for the classification of localizations in SMLM images of sarcomeric proteins. Sarcomeres are highly ordered molecular assemblies, where the order can be exploited to precisely determine the location of proteins by structure averaging^22^. Structure averaging requires the selection of many corresponding regions, which is a time-consuming task if performed manually. Our goal was to address this problem and develop an algorithm to automate the selection of structures to be averaged using the localization coordinates directly. It is worth emphasizing that we did not aim to select only the areas used for averaging; instead, we strived to select the localizations belonging to the structures we were looking for, in other words, our goal was to filter out from the averaging process localizations that do not form part of the structure. Here we present that our machine learning framework provides an efficient implementation of this task. The algorithm not only gives the location of the regions of interest with high efficiency, but also determines which localization within that area belongs to the structure to be analyzed. The developed workflow has been also validated with previously published manual evaluation results^22^. We argue that the work presented here can be generalized as a localization classification workflow that can be used to analyze SMLM images of other structure types too.

## Results

### Training data generation

**Figure 1:**
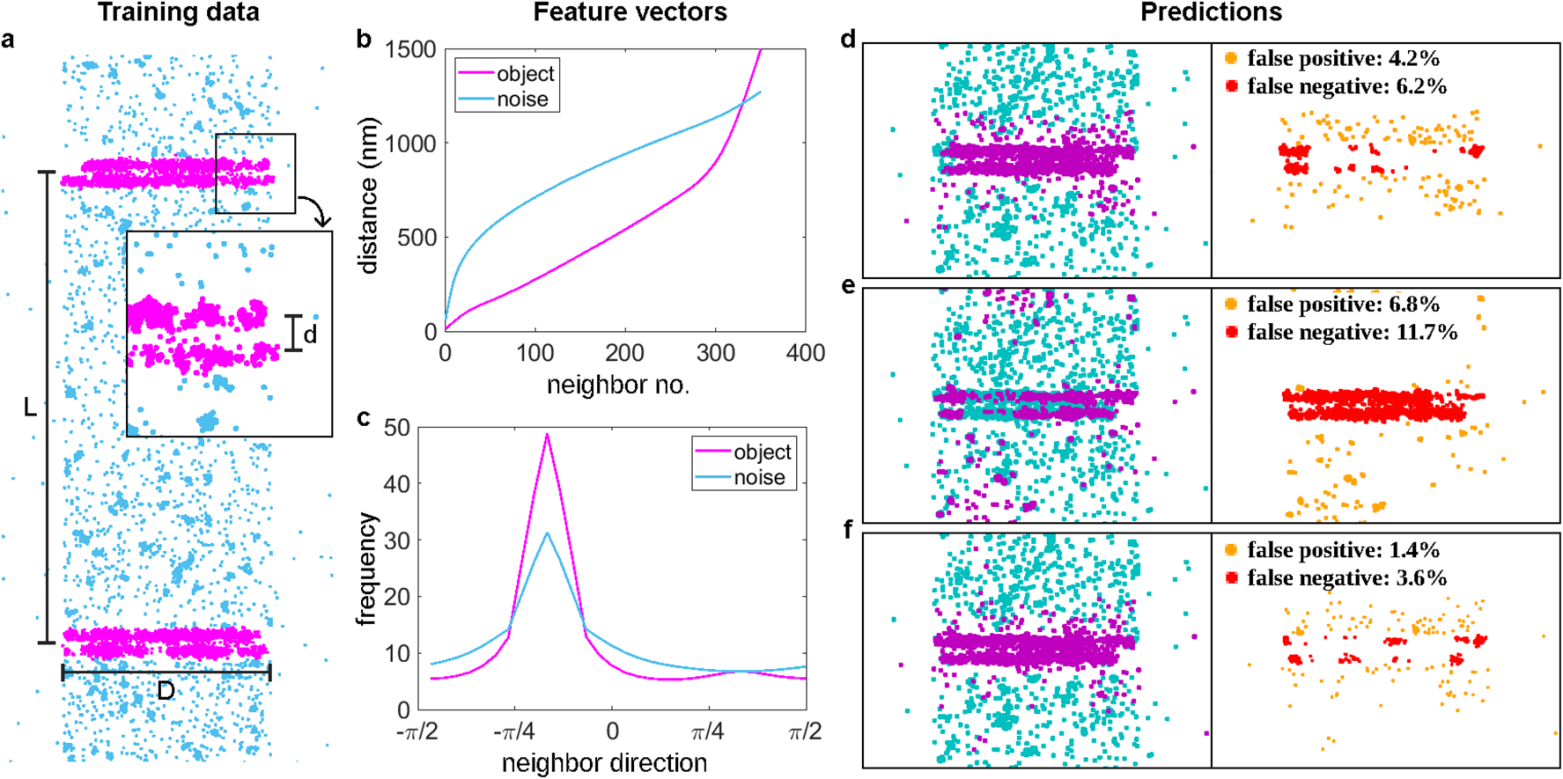
Training data and performance evaluation using different feature vectors. (**a**) Labelled training data generated with TestSTORM (magenta: double-line localizations, cyan: noise points) and the quantities that characterize the structure ($L = 3.4\,\upmu \textrm{m}$, $D = 1.5\,\upmu \textrm{m}$, *d* = 120–128 nm). (**b**,**c**) Average feature vector values for double lines and noise localizations computed from the training data for nearest-neighbor distances (**b**) and for the smoothed distribution of the directions of the neighbors (**c**). (**d**–**f**) The result of neural network classification trained on different feature vectors for one simulated double-line object using the nearest neighbor distances (**d**), the neighbor directions (**e**) and both the distances and directions (**f**). On the left, the result of the classification (dark magenta: localizations classified as part of a double-line object, dark cyan: localizations classified as noise) and on the right, the misclassified localizations (yellow: noise localizations classified as double-line object points, red: double-line localizations classified as noise).

Supervised machine learning requires large amounts of labelled data. Since localization microscopy measurements are time-consuming, expensive and typically lack the knowledge of the ground truth, it is advantageous if simulations can be used to generate training data of sufficient quality. Fortunately, several simulation software packages (SuReSim, FluoSIM, SMeagol, ThunderSTORM, TestSTORM) have been developed in recent years to study imaging artifacts, validate new algorithms or generate training datasets^18,23–26^. We used the TestSTORM test sample generator^27–29^ to generate the labelled training data. In addition to faithfully mimicking the measurements, the tool also ensures that various sample, fluorescence labelling or imaging system parameters can be changed at will. We simulated the dSTORM measurement of epitopes arranged in discs close to each other along a cylinder, since many sarcomeric proteins are arranged in this structure. These cylinders, which correspond to the myofibrils, lie on the cover slip during the measurement, perpendicular to the optical axis, so the 2D dSTORM image of the epitopes gives double-line patterns. Such patterns can be characterized by three quantities: the distance between the lines in the double-line objects (*d*), the distance between the double-line objects on the sarcomere strand (*L*) and the diameter of the disc-shaped structures forming the lines (*D*) (Fig. 1a). The approximate values of these dimensions were already known from previous measurements^22^, so these values were set accordingly in the simulations ($L=3.4\,\upmu \textrm{m}$, $D=1.5\,\upmu \textrm{m}$, and the value of *d* was around 120 nm, depending on the protein) (Table 3). The simulated labelling and acquisition parameters were also set similarly as in the previous measurements (Tables 4, 5), and each generated image stack was evaluated with rainSTORM^30,31^. SMLM images of double lines and non-specific labels or noise were generated separately with varied densities and the localized coordinates were merged in such a way that noise localizations within 50 nm of the double lines were filtered out, as they would have been indistinguishable from the specific labelling and would have confused the training process. The dataset used for training had 626,347 double-line localizations and 509,831 noise points. During training, 10% of these data were used as test data and another 10% were used for validation.

### Calculation of feature vectors

Feature vectors had to be assigned to each localization to provide intput to the neural network for the classification. These vectors have a fixed length and should characterize the localizations well. Williamson at al.^18^ used the distance of the first *N* nearest neighbors as the neural network input to identify cluster points. This work suggests that for a sufficiently accurate output, the last few elements of the vector need to be distances to noise points, which are localizations beyond the cluster boundary. Hence, the whole structure has to be encoded in the elements of the vector. However, this approach raised a problem in our case, as there were significant differences in the number of double-line localizations for different proteins. Consequently, for certain *N* values there were no noise points between the neighbors in relation to which we measured the distances in one dataset, while using the same *N* values in other measurement files the furthest neighbors were already localizations to adjacent double lines. In this way, the localization density of double-line objects significantly modified the form of the feature vectors associated with the localizations, which made training difficult. The goal was to create feature vectors belonging to double-line localizations which always include the distances to all elements of the actual double lines with some noise point distances at the end of the vector values, while keeping the length of the vector fixed. We also aimed to keep the number of computed distances as small as possible to reduce the learning time of the neural network. To solve this problem, we set the length of the vector (e.g. 350) and then divided the localizations into random subsets in which each double-line structure had approximately 300 localizations (Fig. 1b). In these datasets, we calculated the values of the vectors and used these vectors to classify the localizations and then summarized the results. We also took into account that the actual structure is dimensionally anisotropic, i.e. it has a preferred orientation. So the direction of the nearest neighbors also provides valuable information about whether the point is part of a double-line object or a noise point. Therefore, we also determined the distribution of the *N* nearest neighbor directions using 12$^{\circ }$ ($2\pi /30$) sampling intervals (Fig. 1c). This distribution was smoothed with a kernel of size of 3 bins so that noise on the elements of the vector would not interfere with the training. It can be observed that there are two peaks in the averaged angular distribution of the neighbors of the localizations belonging to the double-line object, a characteristic and a weak one, with 90$^{\circ }$ relative orientation. These are the two preferred directions resulting from the structure. The main peak is due to the fact that the neighbors of a localization belonging to double lines are mostly localizations belonging to the same double lines with a specific orientation. The smaller peak is from localizations outside the double lines, which are mainly located along the sacromeric strand, which also has a specific orientation, perpendicular to the orientation of the double lines. The angular distribution of the neighbors of noise points shows only one peak, which is wider than the one for the double-line localizations. This follows from the fact that the neighbors of a noise point will also eventually be densely spaced double-line elements located roughly in the same direction. However, as the reference point is not part of the double lines or is less likely to be located in the line defined by the double-line object, the deviation of direction of these localizations will be larger. Since the direction can vary from structure to structure, we have shifted the global maximum of all distributions to an arbitrarily chosen value. In this way, the values of the feature vectors do not depend on the orientation of the sarcomere. We chose $-\pi /6$ for this arbitrary value, so that the characteristic main and minor peaks in the distributions do not fall on the edge of the distribution curve.

### Performance evaluation

To construct the neural network to perform the localization classification task, we used Keras^32,33^. It is a popular open source machine learning framework, which is easy to use but also provides great flexibility. We used an ANN with one hidden layer, and with this architecture the learning time was only a few hours (without GPU). This three-layer construction performed well when we used only the distance of adjacent localizations as input (95.8% true positive and 93.8% true negative), but had two weaknesses. First, localizations at the ends of the double lines were less likely to be classified as object elements, yielded shorter lines (Fig. 1d, first inset, the left ends of the lines are cyan). The reason of this shortening is that most of the localizations belonging to double lines are densely surrounded by other localizations, while localizations at the ends of the lines are not surrounded as densely by other localizations, and the distance between nearest neighbors starts to shift towards larger values. Another weakness was that noise points adjacent to double lines were easily assigned to the double-line object (Fig. 1d, second inset, yellow dots). The neural network that used only the distribution of the directions of the neighbors performed worse (93.2% true positive and 88.3% true negative), but it was more efficient at finding object localizations at the ends of the double lines than in the middle (Fig. 1e). It was also more efficient at classifying localizations adjacent to double lines as noise. These observations make sense if we consider that at the ends of the lines, the directions of the nearest neighbors are more characteristic around a given angular value, which is a strong indicator for the classification, while towards the edge of the lines the angular distribution of the neighbors’ directions broadens rapidly. Interestingly, the two vectors exactly compensate for each other’s weaknesses. Therefore, their combination could be used to create a feature vector to improve the performance of the neural network. For this, the vector containing the distances of 350 neighbors was concatenated with the 30-element vector containing the distribution of the directions of the neighbor localizations. Thus, a feature vector of size $380 \times 1$ was assigned to each localization. In this way, we achieved a classification accuracy of 98.6% true positive and 96.4% true negative (Fig. 1f). In addition, as the traning process takes less than a day using only CPU, while segmentation and classification of a single double-line structure takes around one minute, we can conclude that this approach is a robust and highly efficient way to reveal the double-line structures.

### Validation by experimental data

To validate our method for the segmentation of sarcomeric structures in the localization data, we had to run the created algorithm on a series of measurements of sarcomeric proteins in which we had previously determined the distribution of epitopes by manually segmenting the double-line objects^22^. From the measurements, we selected three proteins that were stained with varying quality, Capping protein alpha (Cpa), Tropomodulin (Tmod) and Projectin (P5). Of these, the Cpa antibody provided the lowest quality images, where the double lines were almost unresolvable. In contrast, the two lines could be clearly separated in the case of P5, while Tmod staining represents an average quality. Thus, the samples were selected with the goal to test the performance of the algorithm on low, average and high quality localization datasets. Visual comparison of the captured images and the resulting dataset of the three proteins can be found in the supplementary Information (Fig. S1). However,the problem with real measurement files that we do not know how many subsets of the data need to be created so that each subset contains the right number of localizations per double-line object (in our case, ∼ 300 localizations per double-line object). The problem is further complicated by the fact that there can be significant differences between the number of localizations per double-line object even in a single measurement file (partly because of the non-uniform excitation beam during the dSTORM measurement). Therefore, first we had to determine the number of localizations belonging to each individual double-line object, so that they could be partitioned into a sufficient number of random subsets to properly classify the localizations.

### Finding ROIs in measurement files

**Figure 2:**
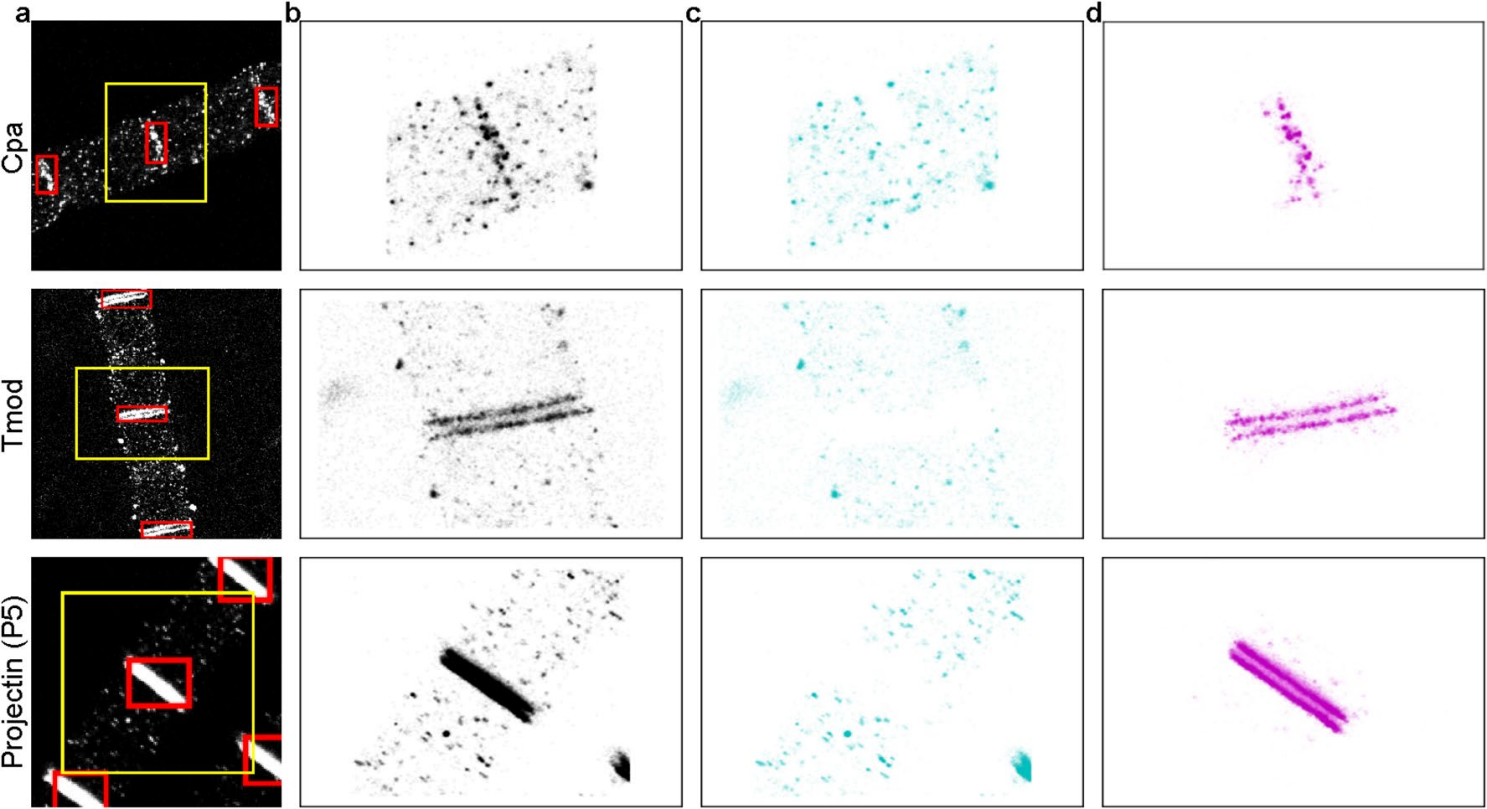
The selection of double-line localizations. (**a**) The pixelated images and the detected double-line objects (red boxes) and analyzed areas (yellow boxes) for three different proteins (*Cpa* Capping protein alpha, *Tmod* Tropomodulin and Projectin (P5)). (**b**) Localization coordinates in the analyzed areas are marked with yellow boxes in (**a**). (**c**) Localization coordinates classified by the algorithm as noise in the given areas (yellow boxes in (**a**)). (**d**) Localization coordinates that the algorithm has assigned to double-line objects in the given areas. Coordinates marked with pale violet have been filtered out afterwards.

Instead of the localization coordinates, first the pixelated images were used for segmentation since several efficient object detection algorithms were developed for such image format^34^. A Mask-RCNN algorithm^35^ was trained to detect double-line objects. We used TestSTORM and rainSTORM to generate training data. The training data was augmented by taking random subsets of the generated localizations and rotating the structure around with a 3$^{\circ }$ step interval. Increasing the size of the training data was necessary because while there were plenty of localizations to use for classification training, a single pixelized image can only be generated by using many localizations. We used an already trained model as a starting point for training our Mask-RCNN model to achieve faster convergence^36^. The trained Mask-RCNN could accurately find ROIs with double-line objects in seconds (Fig. 2a, red boxes). Because the detectability of double lines depended on the contrast of the pixelated images and the ideal contrast value varied from image to image, we ran the detection algorithm at several contrast settings and selected the one with the most areas found with the largest ROIs. After identifying the ROIs, we could estimate the number of localizations within these areas, which was a good approximation of the number of localizations associated with the double lines. After that, the detected ROIs were expanded to meet certain conditions. We shifted the boundaries by a micron so that they encompassed the entire object and contained a sufficient number of noise localizations. Then, we divided the localizations within each ROI into subsets, so that there were $\sim \,300$ object localizations per subset (knowing the approximate localization number in the actual double-line object) and at least 350 localizations including noise points, as the neural network required a 350 element vector input. If the latter condition was not met, the size of the ROI was further increased by half micron increments. The yellow squares in Fig. 2a show ROIs extended in this way, and Fig. 2b figures show the localizations within these extended ROIs. Finding the ROIs in the localization data had another advantage apart from creating the appropriate localization subsets. Calculating the feature vectors for each localization coordinate proved to be time-consuming, as a measurement file typically contains hundreds of thousands of localizations with a significant number of noise localizations. The fact that we calculated feature vectors only for localizations in the critical areas significantly reduced the computational requirements.

### Filtering localizations

After creating the corresponding localization subsets, the feature vectors were calculated for each localization inside the determined regions (marked with yellow squares in Fig. 2a) and the localizations were classified using the trained neural network. Fig. 2c shows the localizations classified as noise within the given areas. Note that in some cases, the extended ROIs that enclose the double-line objects may overlap, and in this case a localization may be assigned with a different label in the different ROIs. One example is the densely packed noise points in the bottom right corner of Fig. 2c Projectin (P5) part. These localizations are classified as double-line elements within another ROI, which included the entire double-line object where these localizations belong to. The object localizations in Fig. 2d were subjected to a follow-up filtering to further reduce the number of false positive localizations. We note that these filtering steps were not performed on the simulated data on which the efficiency of the classification was characterized, but only on the measured datasets. First, we compared the average of the distance of the first 10 nearest neighbors of each double-line element (already calculated for the feature vectors) with the median of the first 10 distances of all double-line elements, which gave a particular value. If this average value was greater than 5 times the median value, the localization was filtered out. This multiplier of 5 was determined from the distribution of the average distances of the first 10 object localization neighbors as a value above which a data point is considered to be a significant outlier. Then, the object localizations were clustered using the DBSCAN algorithm with parameters $\epsilon =50$ nm and $minPts=5$. Using the above-mentioned values for the parameters of the clustering algorithm the localizations of a given double-line object are grouped into one cluster, while neighboring densely packed regions that might be included in the analyzed areas are grouped into separate clusters. From the identified clusters, the cluster with the most localizations was selected as the double-line object. The convex envelopes of the remaining localizations were also determined, as its various parameters (e.g. perimeter, area etc.) are suitable for possible further filtering. After these steps, we did not only define the localizations belonging to the double-line objects, but we also grouped together the localizations belonging to the same object. The classification filtered out a large percentage of localizations (Table 1), while the nearest neighbour and DBSCAN filtering discarded a smaller percentage of localizations. Furthermore, before comparing the results with those obtained by manual segmentation, we applied further strict filtering based on the fitting parameters of the localizations, as we did in the previous work^22^. These included keeping the sigma of the Gaussian curves fitted to the blinking molecules between 112 and 160 nm to filter out localizations from overlapping blinking events and thresholding the calculated Thompson localization precision^37^ with a value of 20 nm. This last filtering step is not strictly part of the classification. The localizations filtered out after all these steps were not used in any further analysis (pale violet localizations in Fig. 2d).

**Table 1:** The ratio of the discarded localization number compared to the total number of localizations in each subsequent filtering step.

	Filtering ratios (%)			
	Classification	Nearest neighbour	DBSCAN	Sigma and precision
Cpa	57.1	0.8	19.5	41.8
Tmod	20.6	0.6	0.1	66.9
Projectin (P5)	42.7	0.5	3.8	58.9

### Comparison with results from manual segmentation

**Figure 3:**
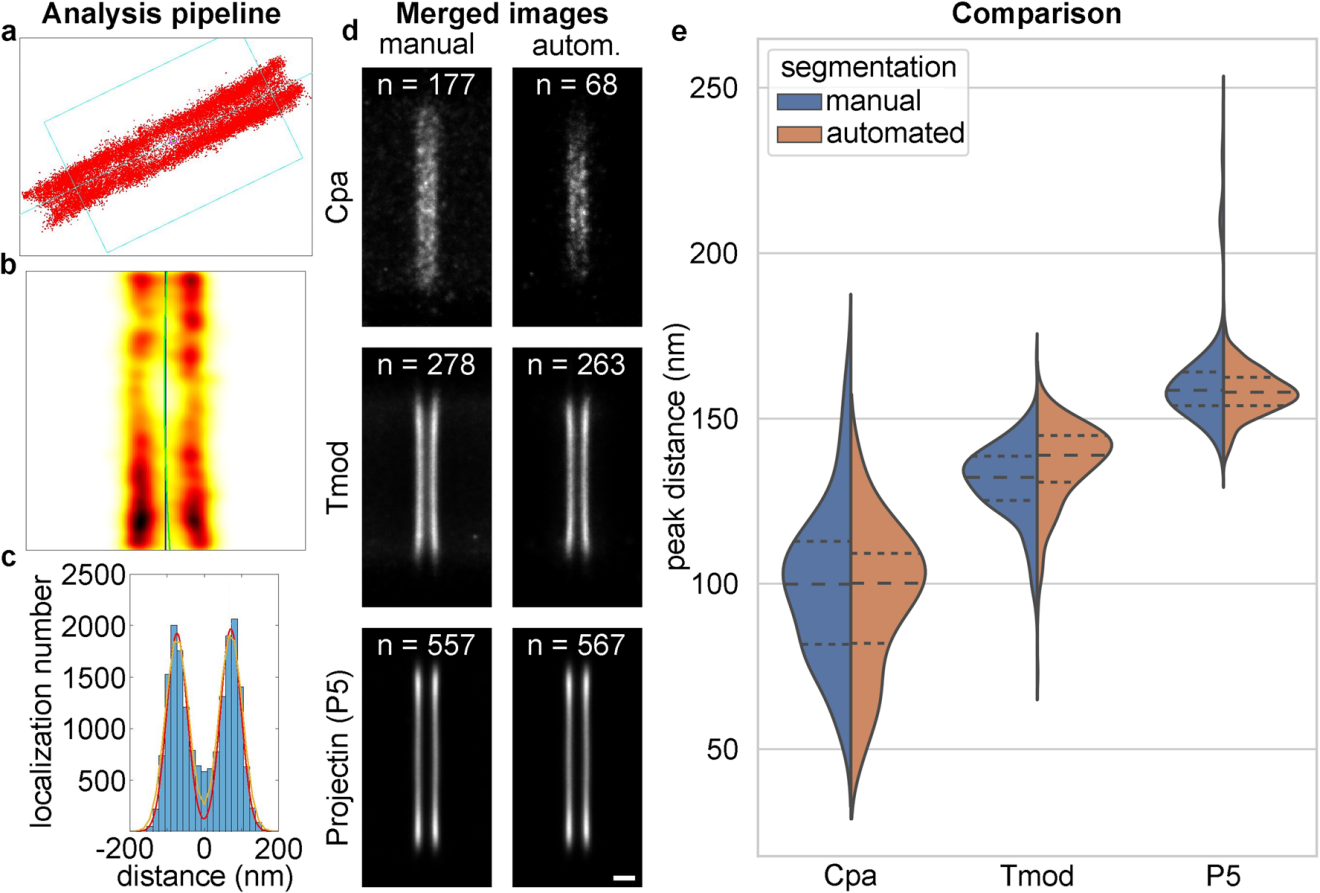
Comparison of the manual and automated segmentation. (**a**) Representation of localizations imported into the IFM-Analyser algorithm. (**b**) Localization density map generated from the imported localizations and the symmetry axis (green curve) fitted to it. (**c**) Distance of the localizations from the symmetry axis and the curve fitted to the distribution (yellow curve) and its correction (red curve). (**d**) Spatial distribution of three proteins labelled with different quality after the structural averaging of dSTORM images for manual and automated segmentation (*Cpa* Capping protein alpha, *T*mod Tropomodulin and Projectin (P5)). (**e**) Comparison of the measured relative positions of proteins for manual and automated segmentation. Scale bar 200 nm (**d**).

On the resulting object coordinates, we ran the IFM-Analyser algorithm^38^ presented in detail in a previous paper^22^. The selected double-line localizations were imported into the IFM-Analyser algorithm (Fig. 3a), where the symmetry axis of the structure was calculated based on the localization density (Fig. 3b). The distribution of the distance of the localizations from the symmetry axis was fitted with a theoretical curve (Fig. 3c, yellow curve), which was corrected considering the localization precision and the size of the antibodies used for labelling (Fig. 3c, red curve). Finally, the distance between the peaks of the resulting curve was determined, which can be used to assess the relative position of the sarcomeric proteins. The merged images of the output double-line objects are shown in Fig. 3d. In case of poorer labelling quality (Cpa), the automated algorithm found only the areas where labelling was best, as can be seen from the number of areas (*n*) found and shown in the figure. In addition, in these areas it tended to classify localizations only in the middle part of the lines as double-line elements. The merged image is therefore shorter but the lines have a higher contrast than with manual segmentation. In the case of Tmod, which had denser labelling, the automated algorithm recognized slightly fewer double-line objects than the manual segmentation, but the difference was not significant. It was observed that in the merged image obtained with the automated algorithm, not only the background disappeared around the lines, but also the area between the lines turned darker, suggesting that the algorithm successfully filtered out the noise localizations between the lines (Fig. 2: Tmod, Supplementary Fig. S2; Table S1). In the case of the Projectin protein, the automated algorithm found more areas as double lines than the manual segmentation method. This is likely the result of the fact that this labelling was of good quality, where the user selected the most visible double-line areas, while the automated algorithm selected areas of interest in a more unbiased manner. We found that the evaluation of the automatically segmented double lines gave very similar results for the relative position of the selected proteins compared to the manually selected regions, validating the efficiency of our method (Fig. 3e, Table 2). The distributions differed the most for the Tmod protein, but even in this case 5.5 nm and 6.7 nm differences were realized between the mean and the median values, respectively. It must be noted that the line separation predicted by the automated algorithm may be closer to the true value, since it successfully filtered out localizations between the lines. Although these localizations do not belong to the real structure, they can pull the peaks closer to each other in the theoretical curve fitting in the IFM-Analyser program. However, the error is in the range of the standard deviation of the measured double-line distances, therefore it does not affect the conclusions of our previous works.

The structure of some sarcomeric proteins has radial dependence. They can form divergent (Tropomyosin (MAC 141), SALS) or convergent (Zormin (B1), Cpa) double lines^22^. The lateral extension can also differ: for example, Sls700 (B2) is localized only in the central region, while others form structures with the same diameter as the sarcomere itself. We believe that these features are not imaging or reconstruction artifacts but depict the real shape of the structures. The three proteins depicted in Fig. 3 were selected to demonstrate this feature too. Cpa provided the lowest image quality. The applied ML algorithm found fewer areas and it also introduced line shortening, i.e. it classified localizations only in the central region. The quality of the Tmod images was significantly higher but the lines were bending, and their separation at the edge increased by approximately 25% (Fig. S3). Projectin (P5) provided the highest image quality and the edge effect was found to be negligible (Fig. S3). The IFM-Analyser discarded the localizations at the edge of the structure, i.e. did not take into consideration the above mentioned line shortening and bending effects, and hence the final molecular model was only valid for the central part of the sarcomere. With such evaluation processes the manual and machine learning algorithms provide the same final results. The edge effect can be studied when the line shortening effects are negligible, i.e. the image quality is high enough and the strict ML algorithm classifies localizations in the bending areas too.

To sum up, these results demonstrate that we have successfully developed a new analysis pipeline to efficiently recognize and classify sarcomere specific double-line features on SMLM images. Although the main part of this analysis was focused on three protein datasets, to test the general applicability of this approach, we probed our workflow in a few additional cases of double-line distribution (Fig. S4). Moreover, we tested it on another structure of interest, sarcomeric single-lines (Fig. S5). These studies showed that our algorithm performs well in these cases too, indicating that our method is not limited to a few proteins and it is suitable for the detection of various types of sarcomeric structures. We believe that our method may have widespread applications and it can be used to recognize similar structures without major modifications.

**Table 2:** Statistical characteristics of the distribution of relative protein positions (*Cpa* Capping protein alpha, *Tmod* Tropomodulin and Projectin (P5)) determined by IFM-Analyser for manual and automated segmentation.

	Peak distances (nm)					
	Cpa		Tmod		Projectin (P5)	
	Manual	Automated	Manual	Automated	Manual	Automated
Mean	99.93	95.56	130.95	136.45	161.55	158.05
Median	99.93	100.19	132.25	138.95	158.55	158.00
Standard deviation	23.45	21.81	11.26	12.43	15.62	7.03

## Discussion

In this study, we constructed, trained and tested a machine learning approach to classify localizations and automatically segment sarcomeric proteins in single-molecule localization microscopy measurements. We used the Mask-RCNN algorithm to find the areas with double-line structures and a neural network to classify the localizations inside the selected areas. Using a fixed-size sequence of input values generated from the distances and directions of the nearest neighbors of each localization as input to the neural network, it was able to identify features within these sequences, which allowed the network to determine with a high degree of confidence whether or not the localizations were part of the double-line objects. These models were trained on data generated with the TestSTORM simulator. This work has shown that training data generated by a simulation software is of satisfactory quality for these kinds of tasks and that neural networks trained with such data are efficient and robust for real measurement data analysis even with large variations in the labelling quality, which we validated with previous measurements. The presented method has proven to be an efficient automation tool for segmenting double-line structures in localization data. A natural extension of this work could be the classification of localizations belonging to other structures. SMLM simulators can be used to generate an arbitrary amount of labelled training data corresponding to a given problem. The generated training data can then be used to search for feature vectors that characterize the localizations well. We assume that the distance to nearest neighbors and their angular distribution is a good starting point for generating this input data. With the right input, the presented neural network can be used to identify other types of structures too.

## Methods

### Experimential details

In this work, previously captured and analyzed dSTORM measurements were used. The generation of the experimental dataset was previously described in detail^22,39^. In brief, we isolated individual myofibrils from the indirect flight muscles (IFM) of Drosophila, and after standard fixation, we performed conventional immunofluorescent labelling. We used dSTORM imaging to measure protein localization in subdiffraction-sized compartments of the IFM sarcomeres, i.e., the H-zone and I-band. These measurements were performed on a custom-made inverted microscope based on a Nikon Eclipse Ti-E frame. EPI illumination at an excitation wavelength of 647 nm ($P_{max}$ = 300 mW; MPB Communications) was applied. An additional laser (405 nm, $P_{max}$ = 60 mW; Nichia) was used for reactivation. Two separate fluorescence filter sets (LF405/488/561/635-A-000 and Di03-R635-t1 dichroic mirrors with BLP01-647R-25 emission filters; Semrock) were used to select and separate the excitation and emission lights in the microscope. For the imaging, oil immersion objective (CFI Apo $100\times$, 1.49 NA; Nikon) was used. Typically 20,000–50,000 frames were captured with an exposure time of 20 or 30 ms by an Andor iXon3 897 BV EMCCD digital camera ($512 \times 512$ pixels with $16\, \upmu \textrm{m}$ pixel size). We acquired 2D projections of the myofibrils, which revealed the lateral protein distribution patterns at the H-zones and I-bands. We classified these patterned structures as double lines, single lines, or gaps. We processed the measurements with rainSTORM, and performed drift correction and filtering. After quality control, the structures were manually selected and the raw localization information of the selected structures was exported for further analysis. Using IFM Analyser^22,39^, we measured the epitope distribution along the longitudinal axis of myofibrils and for visualization purposes, we aligned the localizations along the symmetry axes of the H-zone/I-band by rotation and translation and generated averaged images. We analyzed the localization of 14 epitopes in the H-zone and 21 epitopes in the I-band, using dSTORM images of $\sim \, 9000$ sarcomeres. To test our ML approach, we selected 3 epitopes/proteins (Cpa, Tmod, Projectin (P5)) with different labelling qualities and used the corresponding raw datasets. To get an overview on the full process of the evaluation of the measurement datasets using the automated segmentation, see Fig. S6.

### Simulation details

To simulate the sarcomeric double lines, synthetic image stacks were generated with TestSTORM using the ‘Disks’ pattern. This pattern consist of parallel disc pairs, whose projections form double-line structures. Binding sites for the dyes are placed on both sides of the discs with varying density. Each simulation contained 27 double lines. The noise localizations were simulated by TestSTORM’s randomly placed ‘Vesicles’ patterns with a single binding site and with the ‘Non-specific labels’ functionality of the software. While the former patterns were confined to a 600 nm thick volume around the focus, and each vesicle resulted in a localization cluster, the non-specific labels were generated randomly in a 7 confocal parameter thick volume and provided more evenly distributed localizations. Simulation parameters were matched to the experimental parameters (Tables 3, 4, 5). The localization dataset of noise/non-specific labelling and double-line objects were generated separately to enable their separation during training. The simulated image stacks were evaluated with the rainSTORM localization software with the default settings, and the obtained localization datasets were used for the training.

**Table 3:** Characteristics of simulated patterns in TestSTORM.

Sample parameters	
Noise as randomly distributed small vesicles	
Maximum dz (nm)	300
Radius of vesicles (nm)	0
Number of epitopes	1
Double-line objects as disks along a cylinder	
L (nm)	3400
D (nm)	1500
d (nm)	120–128
Dens (1/$\upmu \textrm{m}^2$)	30–50

**Table 4:** Used labeling parameters in TestSTORM.

	Labelling parameters	
	Double-line objects	Noise
Emission WL (nm)	665	665
Char. ON time (s)	0.03	0.05
Char. OFF time (s)	50	42
Bleaching constant (s)	1700	1700
Emitted photon/s	80,000	100,000
Mean bonding angle ($^\circ$)	0	0
SD of bonding angle ($^\circ$)	30	30
Mean N of labels/epitope	5, 6	4, 6
Var. N of labels/epitope	2	0, 1, 2
Length of linkers (nm)	25, 30	10, 15, 30
Non-spec. l. dens. ($1/\mu \textrm{m}^3$)	0	0, 3

**Table 5:** Used acquisition parameters in TestSTORM.

Acquisition parameters	
Number of frames	15,000–20,000
Frame rate (1/s)	20
Exp. time (s)	0.02
Pixel size (nm)	160
Av. BG level	200
RI of immersion m.	1.518
RI of sample m.	1.331
Numerical aperture	1.4
Electron/count	21.5
Pre-amplification	2.5
Actual EM gain	90
Quantum efficiency	0.9

### Neural network characteristics

The Artificial Neural Network (ANN) used for the classification of the data points contained one hidden layer with 32 units using the ReLU activation function as non-linearity. The loss function was binary cross-entropy with a single sigmoid output neuron. Adam was used as an optimizer. The model was trained for 10 epochs with a batch size of 32. Implementation and training was completed in ‘keras 2.2.4’ using a consumer grade Intel Core i7-6700K CPU without utilizing any GPU. With such a setup training takes less than a day. Classification roughly scales with N*log(N) on large datasets, bounded by the k-nearest neighbor algorithm. The classification of a single double-line structure takes around one minute on a regular dataset, while the object detection of a dataset contaning $\sim \,20$ doubleline structures requires less than a minute.

## Supplementary Information

Supplementary Information.

## Data Availability

The code can be downloaded from a public Gitlab repository^40^. The training dataset is available on Zenodo^41^. The datasets used and analyzed during the current study are available from the corresponding author upon reasonable request.

## References

[1] Betzig E (2006). Imaging intracellular fluorescent proteins at nanometer resolution. Science.

[2] Hess ST, Girirajan TP, Mason MD (2006). Ultra-high resolution imaging by fluorescence photoactivation localization microscopy. Biophys. J ..

[3] Rust MJ, Bates M, Zhuang X (2006). Sub-diffraction-limit imaging by stochastic optical reconstruction microscopy (STORM). Nat. Methods.

[4] Heilemann M (2008). Subdiffraction-resolution fluorescence imaging with conventional fluorescent probes. Angew. Chem. Int. Ed..

[5] Huang B, Babcock H, Zhuang X (2010). Breaking the diffraction barrier: Super-resolution imaging of cells. Cell.

[6] Endesfelder U, Malkusch S, Fricke F, Heilemann M (2014). A simple method to estimate the average localization precision of a single-molecule localization microscopy experiment. Histochem. Cell Biol..

[7] Lelek M (2012). Superresolution imaging of HIV in infected cells with flash-palm. Proc. Natl. Acad. Sci..

[8] Szymborska A (2013). Nuclear pore scaffold structure analyzed by super-resolution microscopy and particle averaging. Science.

[9] Salvador-Gallego R (2016). Bax assembly into rings and arcs in apoptotic mitochondria is linked to membrane pores. EMBO J..

[10] Mund M (2018). Systematic nanoscale analysis of endocytosis links efficient vesicle formation to patterned actin nucleation. Cell.

[11] Andronov L, Ouararhni K, Stoll I, Klaholz BP, Hamiche A (2019). Cenp-a nucleosome clusters form rosette-like structures around hjurp during g1. Nat. Commun..

[12] Owen DM, Gaus K (2013). Imaging lipid domains in cell membranes: The advent of super-resolution fluorescence microscopy. Front. Plant Sci..

[13] Griffié J (2018). Dynamic Bayesian cluster analysis of live-cell single molecule localization microscopy datasets. Small Methods.

[14] Wu Y-L, Tschanz A, Krupnik L, Ries J (2020). Quantitative data analysis in single-molecule localization microscopy. Trends Cell Biol..

[15] Auer A, Strauss MT, Strauss S, Jungmann R (2020). Nanotron: A picasso module for mlp-based classification of super-resolution data. Bioinformatics.

[16] Heydarian H (2018). Template-free 2d particle fusion in localization microscopy. Nat. Methods.

[17] Jordan MI, Mitchell TM (2015). Machine learning: Trends, perspectives, and prospects. Science.

[18] Williamson DJ (2020). Machine learning for cluster analysis of localization microscopy data. Nat. Commun..

[19] Khater IM, Meng F, Wong TH, Nabi IR, Hamarneh G (2018). Super resolution network analysis defines the molecular architecture of caveolae and caveolin-1 scaffolds. Sci. Rep..

[20] Khater, I. M., Hamarneh, G., Nabi, I. R. & Meng, F. Caveolin-1 domain characterization using machine learning and graphlet analysis of single molecule super resolution microscopy. In *MOLECULAR BIOLOGY OF THE CELL*, vol. 29 (AMER SOC CELL BIOLOGY 8120 WOODMONT AVE, STE 750, BETHESDA, MD 20814-2755 USA, 2018).

[21] Khater IM, Aroca-Ouellette ST, Meng F, Nabi IR, Hamarneh G (2019). Caveolae and scaffold detection from single molecule localization microscopy data using deep learning. PLoS One.

[22] Szikora S (2020). Nanoscopy reveals the layered organization of the sarcomeric h-zone and i-band complexes. J. Cell Biol..

[23] Venkataramani V, Herrmannsdörfer F, Heilemann M, Kuner T (2016). Suresim: Simulating localization microscopy experiments from ground truth models. Nat. Methods.

[24] Lagardère M, Chamma I, Bouilhol E, Nikolski M, Thoumine O (2020). Fluosim: Simulator of single molecule dynamics for fluorescence live-cell and super-resolution imaging of membrane proteins. Sci. Rep..

[25] Lindén M, Ćurić V, Boucharin A, Fange D, Elf J (2016). Simulated single molecule microscopy with smeagol. Bioinformatics.

[26] Ovesnỳ M, Křížek P, Borkovec J, Švindrych Z, Hagen GM (2014). Thunderstorm: A comprehensive imagej plug-in for palm and storm data analysis and super-resolution imaging. Bioinformatics.

[27] Sinkó J (2014). Teststorm: Simulator for optimizing sample labeling and image acquisition in localization based super-resolution microscopy. Biomed. Opt. Express.

[28] Novák T, Gajdos T, Sinkó J, Szabó G, Erdélyi M (2017). Teststorm: Versatile simulator software for multimodal super-resolution localization fluorescence microscopy. Sci. Rep..

[29] Teststorm. http://titan.physx.u-szeged.hu/~adoptim/?page_id=183 (2021).

[30] Rees EJ, Erdelyi M, Schierle GSK, Knight A, Kaminski CF (2013). Elements of image processing in localization microscopy. J. Opt..

[31] Rainstorm. http://titan.physx.u-szeged.hu/~adoptim/?page_id=582 (2021).

[32] Keras. https://keras.io (2022).

[33] Chollet, F. Keras. https://github.com/fchollet/keras (2015).

[34] Zou, Z., Shi, Z., Guo, Y. & Ye, J. Object detection in 20 years: A survey. arXiv:1905.05055 (arXiv preprint) (2019).

[35] He, K., Gkioxari, G., Dollár, P. & Girshick, R. Mask r-cnn. In *Proceedings of the IEEE International Conference on Computer Vision*, 2961–2969 (2017).

[36] Abdulla, W. Mask r-cnn for object detection and instance segmentation on keras and tensorflow. https://github.com/matterport/Mask_RCNN (2017).

[37] Thompson RE, Larson DR, Webb WW (2002). Precise nanometer localization analysis for individual fluorescent probes. Biophys. J..

[38] Gajdos, T., T. és Novák. https://titan.physx.u-szeged.hu/~adoptim/?page_id=1246 (2022).

[39] Szikora S, Novák T, Gajdos T, Erdélyi M, Mihály J (2020). Superresolution microscopy of drosophila indirect flight muscle sarcomeres. Bio-Protoc..

[40] Varga, T., D. és Novák. https://gitlab.com/adoptim/sarcomere-segmentation (2022).

[41] Varga, T., D. és Novák. 10.5281/zenodo.7306868 (2022).

