# Supplementary material for: Machine learning framework to segment sarcomeric structures in SMLM data
Source: Sci Rep. 2023 Jan 28;13:1582. doi: 10.1038/s41598-023-28539-7 (PMC9884202; doi:10.1038/s41598-023-28539-7)
Supplement: Supplementary file 1 — Supplementary Information. [file 41598_2023_28539_MOESM1_ESM.pdf]

Supplementary Information: „Machine learning framework to segment sarcomeric structures in SMLM data"

*Dániel Varga[1, *], Szilárd Szikora[2], Tibor Novák[1], Gergely Pap[3], Gábor Lékó[4], József Mihály[2, 5], and Miklós Erdélyi[1, **]*

[1]Department of Optics and Quantum Electronics, University of Szeged, Dóm tér 9, Szeged, 6720, Hungary
[2]Institute of Genetics, Biological Research Centre, Temesvári körút 62, Szeged, 6726, Hungary
[3]Department of Computer Algorithms and Artificial Intelligence, University of Szeged, Árpád tér 2, Szeged, 6720,
Hungary
[4]Department of Software Engineering, University of Szeged, Dugonics tér 13, Szeged, 6720, Hungary
[5]Department of Genetics, University of Szeged, Közép fasor 52, Szeged, 6726, Hungary
[*]vdaniel@titan.physx.u-szeged.hu
[**]erdelyi.miklos@szte.hu

Supplementary Figure S1

To test our algorithm we have selected three proteins with significantly different labelling quality. Of those, the Cpa (Fig. S1 a and e) shows the lowest number of and the least specific blinking events, which results in barely recognizable double lines and numerous, clustered noise localizations between them. In case of the Tmod (Fig. S1 b and f), the double are clearly observable, but there are noise localizations between the double-line structures in high density. The P5 (Fig. S1 c and g) provides the highest quality measurement data with clearly formed double lines and minimal noise localizations.

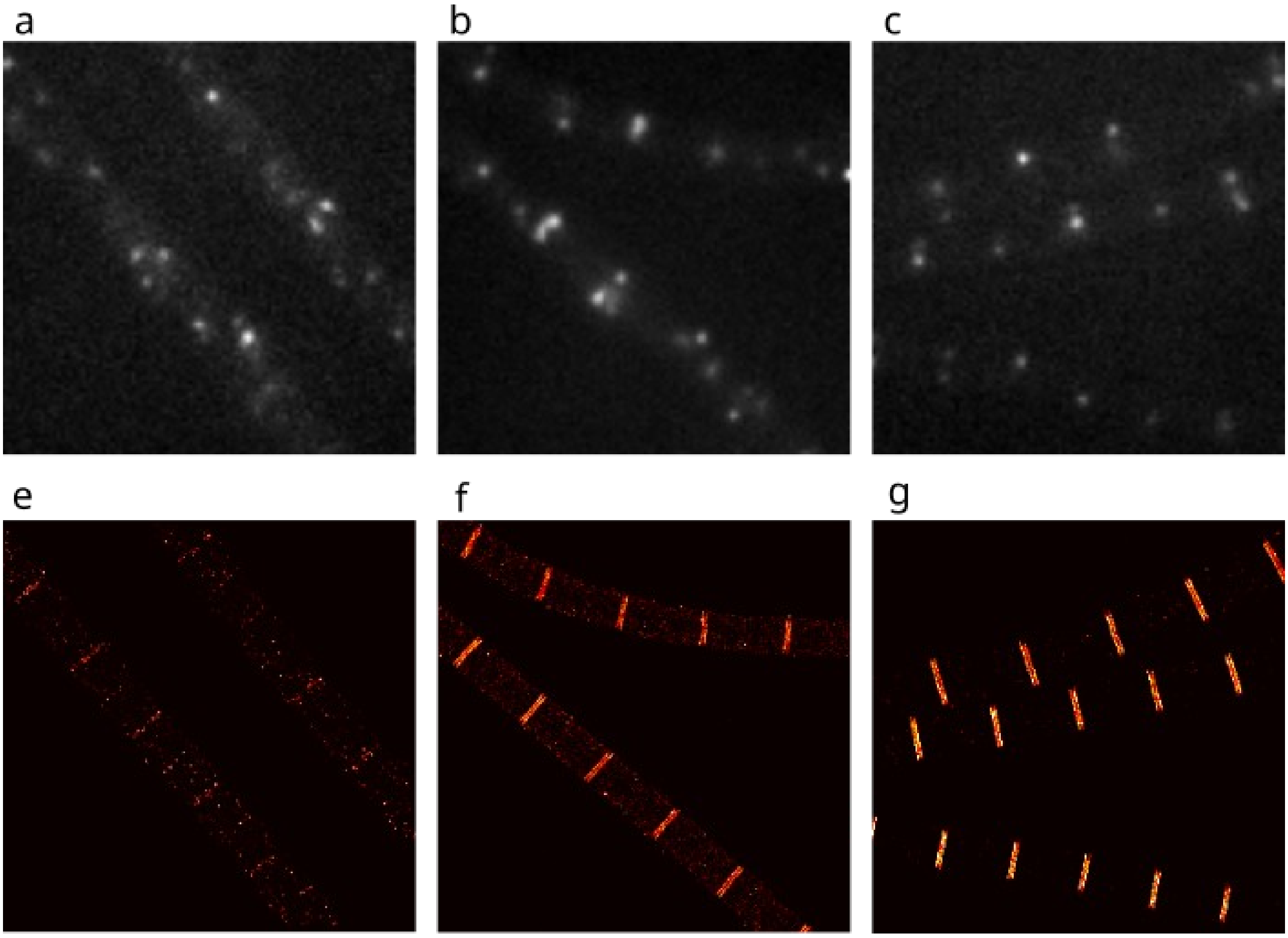

Figure S1: *Representative frames from the Cpa (a), Tmod (b) and P5 (c) dSTORM raw image stacks (with autoscaled contrast) and the resultant super-resolved images (e-g) (with fix contrast scaling), respectively.*

Supplementary Figure S2 and Table S1

Automated evaluation using the machine learning framework resulted in a darker area between the lines in case of the Tmod protein as shown in Fig. 3d in the manuscript. Here we quantify the difference between the manual and automated evaluation techniques.

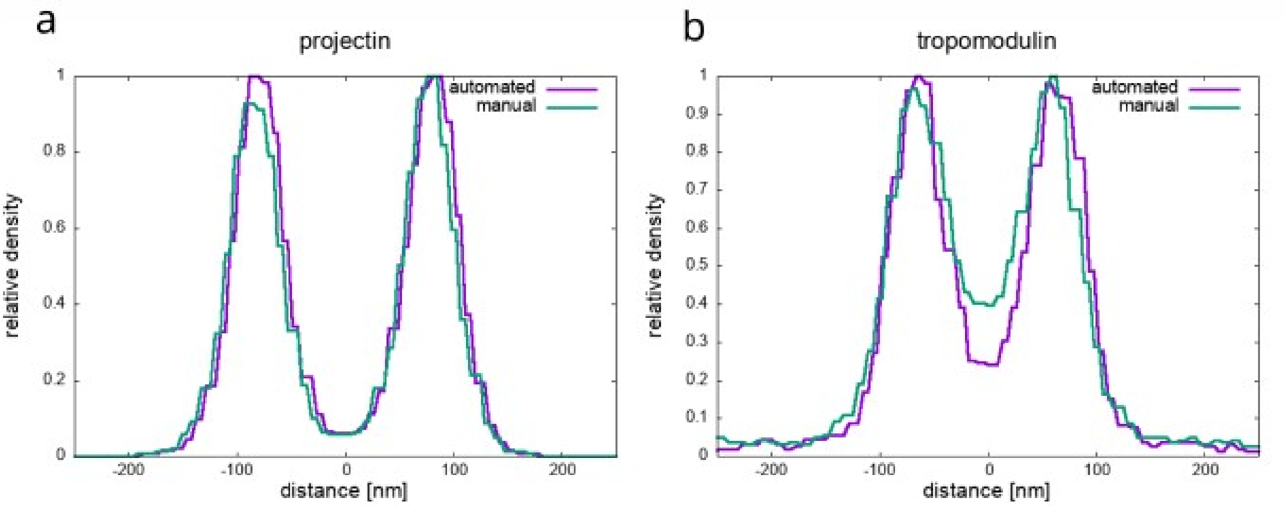

Figure S2: *Cross-section profiles of merged P5 (a) and Tmod (b) structures.*

	P5	Tmod
manual	88.7%	42.3%
automated	88.7%	60.9%

Table S1: *The measured contrast values on the merged figures of the P5 and Tmod proteins.*

The contrast was calculated using the mean height of two peaks and the minimum between the peaks:

$$C=\frac{I_{peak,mean}-I_{minimum}}{I_{peak,mean}+I_{minimum}}$$

Supplementary Figure S3

The double-line structure is not strictly parallel for every protein. Here we quantified the line separation at multiple distances from the sarcomere centre.

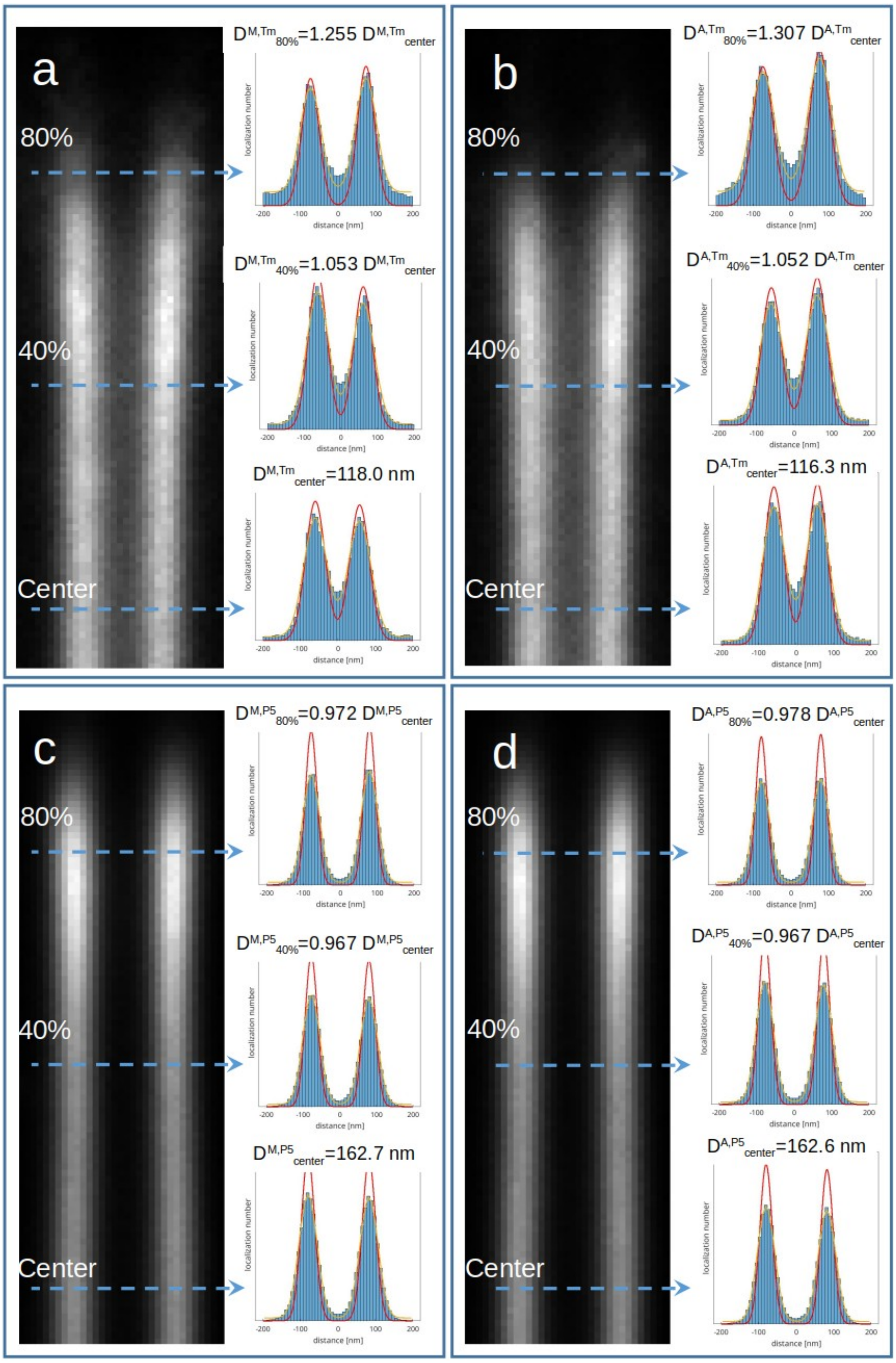

Figure S3: *Separation of Tmod (a, b) and Projectin (P5) (c, d) lines as a function of radial position using the manual (a, c) and the automated, machine learning (b, d) approaches. Both evaluation methods provide the same line bending values. The separation of the Tmod lines increases approximately by 5% and 25% at positions 40% and 80% of the structure, respectively. In contrast no significant line bending effect can be visualized for Projectin (P5).*

Supplementary Figure S4

We have tested the algorithm on additional sarcomeric proteins (SALS, Kettin) providing double-line structures besides the ones (Cpa, Tmod, P5) shown in the manuscript. here we present the comparative results of the automated and the manual evaluation for SALS in the H-zone and for Kettin Ig16 labelled . They indicate that the trained algorithm is not limited to the few proteins we investigated.

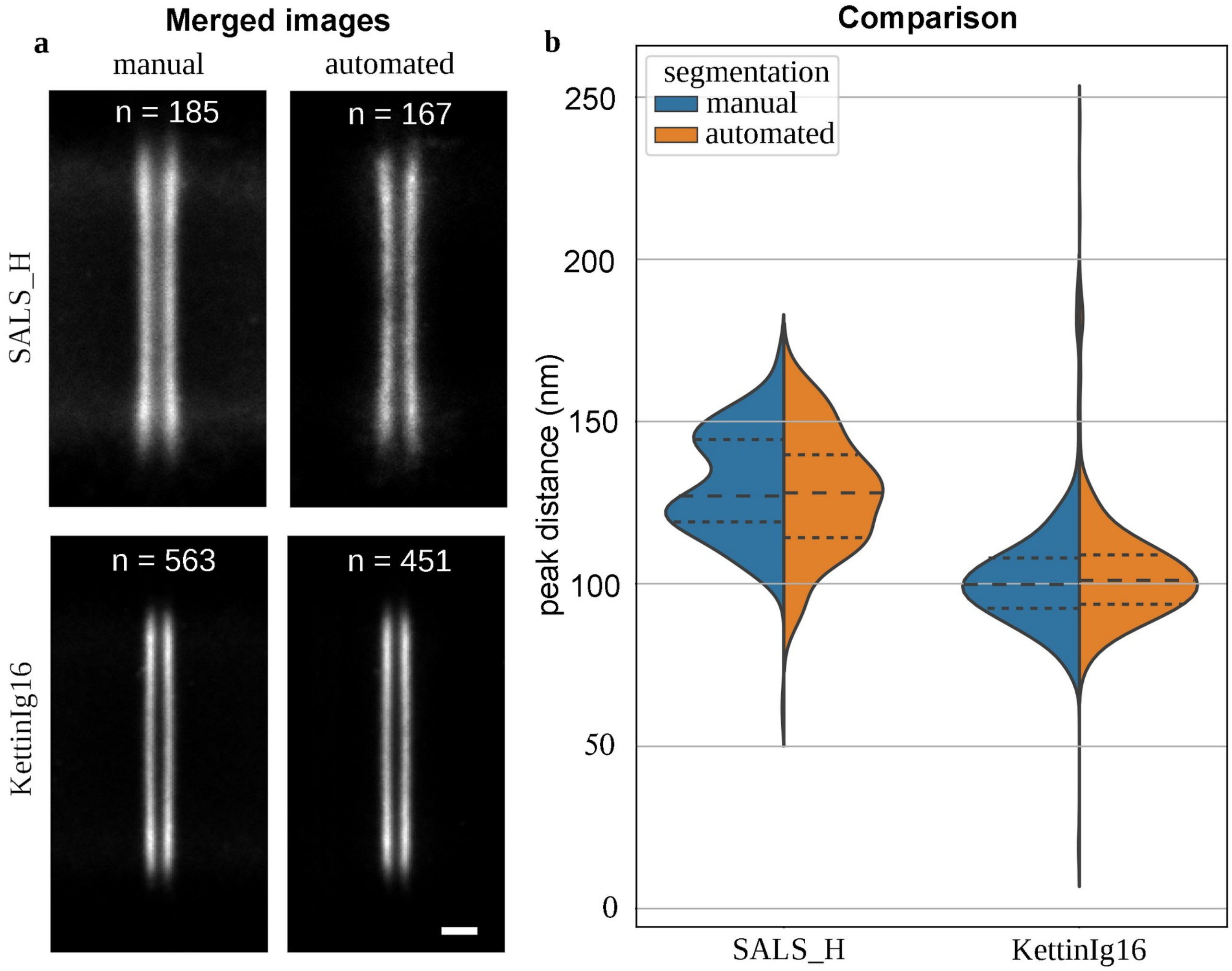

Figure S4: *Comparison of the peak distance results for the SALS_H and the KettinIg16 formed double-lines.*

Supplementary Figure S5

Our algorithm can be used to evaluate measurements performed on structures other than double lines. Here we tested the applicability of the algorithm on sarcomeric single lines formed by the Zasp52 protein in the Z-disks. The Mask-RCNN object detection identified the vast majority of single lines, and the ANN classified the localizations with high accuracy.

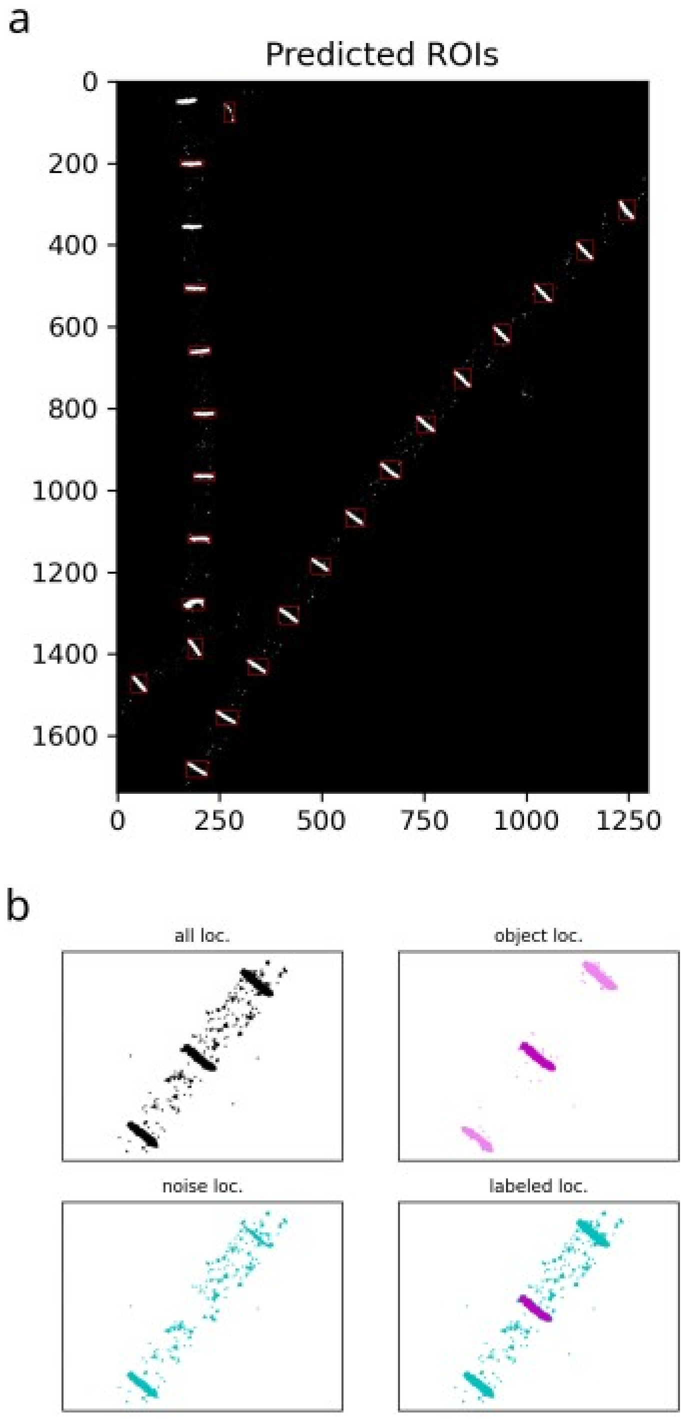

Figure S5: *Segmented single lines formed by Zasp52 in a dSTORM image of two myofibrils (a), and the classified localizations of a selected single line (b).*

Supplementary Figure S6

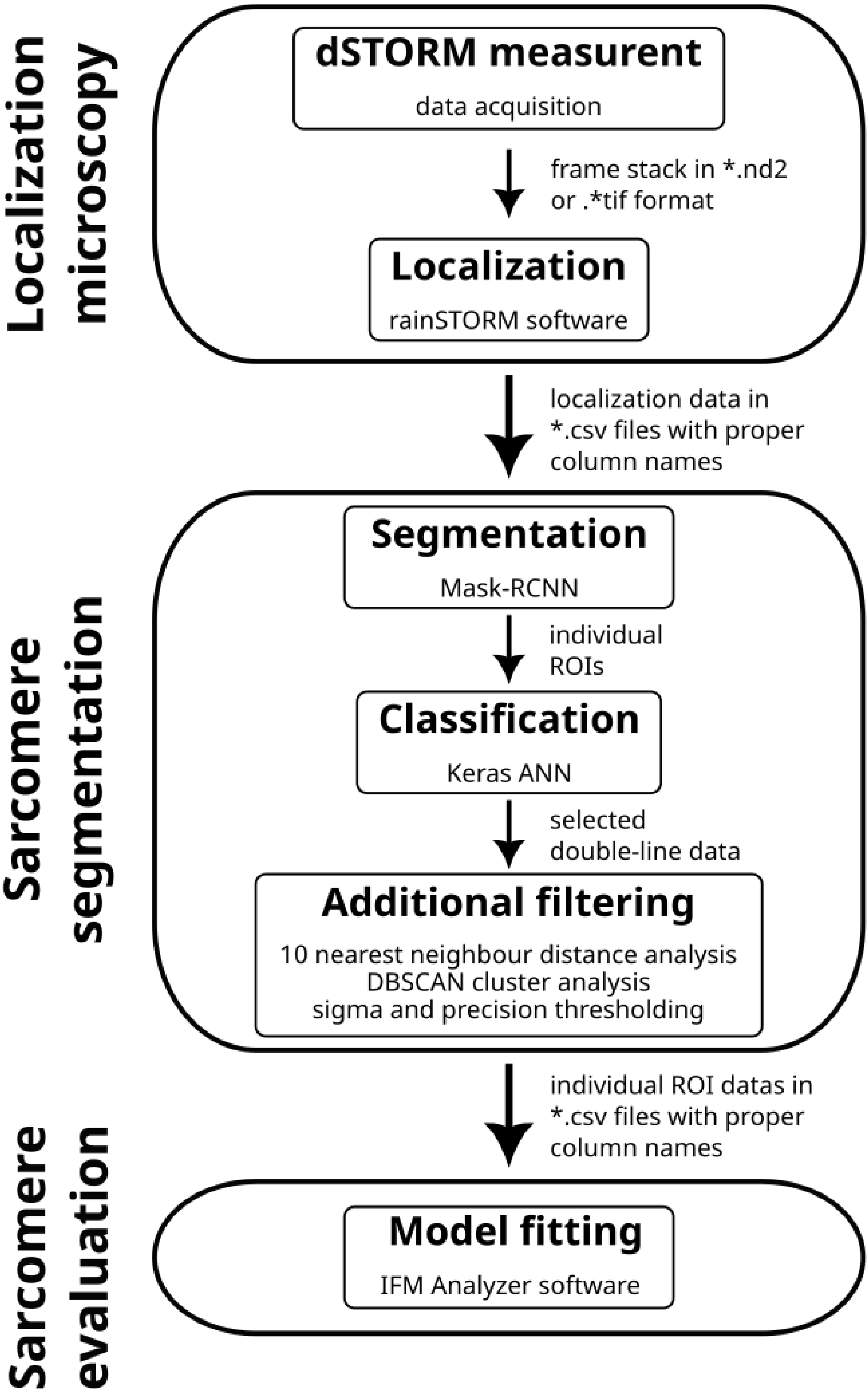

Figure S6: The full process of the evaluation of the measurement datasets using the automated segmentation.
